# A Grey Model and Mixture Gaussian Residual Analysis-Based Position Estimator in an Indoor Environment

**DOI:** 10.3390/s20143941

**Published:** 2020-07-15

**Authors:** Yan Wang, Wenjia Ren, Long Cheng, Jijun Zou

**Affiliations:** Department of Computer and Communication Engineering, Northeastern University, Qinhuangdao 066004, China; neurwj@163.com (W.R.); chenglong@neuq.edu.cn (L.C.); neuzjj@126.com (J.Z.)

**Keywords:** indoor environment, non-line of sight, sequential target tracking, grey model, mixture Gaussian residual analysis

## Abstract

As the progress of electronics and information processing technology continues, indoor localization has become a research hotspot in wireless sensor networks (WSN). The adverse non-line of sight (NLOS) propagation usually causes large measurement errors in complex indoor environments. It could decrease the localization accuracy seriously. A traditional grey model considers the motion characteristics but does not take the NLOS propagation into account. A robust interacting multiple model (R-IMM) could effectively mitigate NLOS errors but the clipping point is hard to choose. In order to easily cope with NLOS errors, we present a novel filter framework: mixture Gaussian fitting-based grey Kalman filter structure (MGF-GKFS). Firstly, grey Kalman filter (GKF) is proposed to pre-process the measured distance, which can mitigate the process noise and alleviate NLOS errors. Secondly, we calculate the residual which is the difference between the filtered distance of GKF and the measured distance. Thirdly, a soft decision method based on mixture Gaussian fitting (MGF) is proposed to identify the propagation condition through residual value and give the degree of membership. Fourthly, weak NLOS noise is further processed by unscented Kalman filter (UKF). The filtered results of GKF and UKF are weighted using the degree of membership. Finally, a maximum likelihood (ML) algorithm is applied to get the coordinate of the target. MGF-GKFS is not supported by any of the priori knowledge. Full-scale simulations and an experiment are conducted to compare the localization accuracy and robustness with the state-of-the-art algorithms, including robust interacting multiple model (R-IMM), unscented Kalman filter (UKF) and interacting multiple model (IMM). The results show that MGF-GKFS could achieve significant improvement compared to R-IMM, UKF and IMM algorithms.

## 1. Introduction

A wireless sensor network (WSN) is an independent self-organized monitoring system. It is composed of many small sensors that are equipped with power supply and radio frequency modules [1]. The sensor nodes are densely placed in unattended detecting regions. The goal of the system is to complete designated monitoring tasks independently. It can be applied in many smart scenarios [2], such as disaster management, medical treatment, space exploration, security defense and so on. Therefore, the emergence of WSN has attracted great attention from military departments, industry and academia in many countries. Meanwhile, the mobile localization technology is a crucial part of WSN because position information is the foundation of applications based on sensor networks [3].

The localization method in outdoor environments is relatively mature. The Global Positioning System (GPS) could provide high accuracy position estimation [4]. However, when the target moves to an indoor environment, the positioning accuracy will reduce sharply due to the deep shadowing of buildings where the signal propagation is blocked [5]. Hence, indoor localization technology based on WSN has aroused wide concerns. The localization system is mainly composed of beacon nodes and mobile nodes [6] while the localization algorithms can be classified as range-based and range-free. For range-based method, beacon nodes with known position send signals to the target in the detecting area and convert the received signals into distance or angle information. The distance or angle values are used to obtain the position estimation of the target through the geometric relationship, such as trilateration, triangulation and multilateration. As for the range-free localization method, we utilize the connectivity of the network to estimate the location roughly, such as centroid algorithm, convex programming and DV-Hop. These methods do not need additional support of hardware so the energy consumption is very low. However, the range-free methods fail when high positioning accuracy is needed.

In the range-based localization system, the practical hardware of beacon nodes can be UWB (ultra-wideband), Bluetooth, wireless local area network (WLAN), etc. These modules adopt different ranging methods which mainly include time of arrival (TOA) [7], roundtrip time of flight (RTOF), time difference of arrival (TDOA) [8], received signal strength (RSS) [9], angle of arrival (AOA) [10] and phase difference of arrival (PDOA). When the propagation path is direct, the environment is line of sight (LOS) and thus we can get accurate position estimation. However, if the radio propagation path is broken by the obstacle, the non-line of sight (NLOS) environment will cause a large deviation to the position estimation, mostly because the reflection, refraction, scattering and diffraction propagation effects caused by the obstacles. How to mitigate or identify the NLOS measurements is still a challenging research direction.

In this article, a mixture Gaussian fitting-based grey Kalman filter structure algorithm (MGF-GKFS) is proposed to tackle this issue. The advantages of our algorithm are as follows:

- GKF is proposed to pre-process the measured distance. It could mitigate the process noise caused by target random moving speed and direction. If the propagation path is NLOS, the adverse distance measurement error interference can also be effectively alleviated. After pre-processing, the localization accuracy significantly improves the IMM algorithm.
- Since GKF greatly reduces noise interference, the residual could be calculated by subtracting the pre-processed value from measurement, which is closer to the true measurement error.
- A soft NLOS identification method is proposed to give the probability of conditions based on residual analysis. Only one measurement needs to be done at a certain time step, instead of taking multiple measurements in a traditional probabilistic weighting algorithm based on a Gaussian mixture model (GMM) [11].
- We adopt a TOA ranging method based on UWB in 2D scenario. The proposed algorithm could apply to other range-based localization model such as RSS and AOA. The entire algorithm is not supported by any priori knowledge.
- Full-scale simulations and experiments are conducted to validate the robustness of our algorithm under various NLOS distributions. An actual scenario experiment proves good performance compared to R-IMM, unscented Kalman filter (UKF) and interacting multiple model (IMM).
- MGF-GKFS is based on the distance filtering so it can be easily extended to 3D scenes.

We organize this article as follows. In Section 2, some key notations are listed. In Section 3, the related works are detailed. In Section 4, we restate the problem and give a brief introduction to GMM. In Section 5, the proposed method is elaborated. In Section 6, the results of simulations and experiments are displayed to illustrate the performance of our algorithm. Finally we reach a conclusion in Section 7.

## 2. Notations

Table 1 lists some of the more frequently used symbols in this article.

## 3. Related Works

The convex optimization problem becomes a challenge for solving node position estimation in a complex NLOS environment. The traditional maximum likelihood (ML) estimation algorithm failed in adverse NLOS condition. To address this problem, numerous mathematical methods were applied to WSN positioning, such as the linear-programming (LP) approach [12], min-max algorithm [13] and robust multilateration algorithm [14]. Furthermore, with exploiting the idea of grouping, a robust estimator has been proposed in [15,16,17] by converting the positioning problem into a generalized trust region sub-problem [9]. This method changes the global convex optimization problem into a non-convex problem for each sub-region so that it can be solved easily by ML. Instead of processing the measured values mathematically [12,13,14,15,16,17], many NLOS mitigation methods have been proposed. In [18], the authors improved the traditional residual mitigation approach [4] through grouping the range measurements to different subsets. In [11], the authors presented a probability weighted fusion algorithm based on GMM. It combines multiple modes in the mixture Gaussian model to update the Kalman parameter. Many intelligent algorithms have also been applied to NLOS mitigation. In [19], a low computational NLOS mitigation algorithm using the sparse pseudo-input Gaussian process has been proposed, which eliminated the deviation by improving existing GP regression methods. In [20], the simulated annealing (SA) algorithm is exploited to optimize the position estimation. However, it is difficult to control the parameters and cannot guarantee the convergence of the objective function. This is a common problem with many intelligent optimization algorithms.

Different from directly mitigating NLOS errors above, many NLOS identification methods have also been proposed. These methods firstly identify which condition the mobile node is in and then use different models to process different conditions. NLOS error identification methods can be divided into two categories: hard decision [21,22,23,24,25] and soft decision [4,6,9]. In [21], a probabilistic location selection method through pedestrian dead reckoning is proposed. The contribution of [21] is that a low-complexity hard decision algorithm is proposed to identify the transformation process of the conditions. Some novel intelligent algorithms have also been applied to NLOS recognition [22,23,24,25]. In order to match standardized fingerprints, a similarity metric, termed the signal tendency index (STI), is established in [23,24]. The disadvantage of them is the poor- adaptability to the new scenarios because the foundation of them is the pre-built fingerprints. In [22], the authors made improvements to support vector machine (SVM) using convolution neural networks. The error covariance matrix could be adjusted automatically according to the adaptive filter. This method has good adaptability to the new environment. Moreover, in [25], the authors proposed the import vector machine. Compared with SVM, the feature selection strategy is used to ameliorate the accuracy of classification. As for soft NLOS identification, in [4], a NLOS clustering algorithm based on fuzzy C-means is proposed to classify the NLOS condition to a soft NLOS condition and hard NLOS condition and also give the degree of membership to each of them. Therefore, the identification of the NLOS environment is still a very open topic. There is no one very common method now. We can adjust the existing method according to our model to obtain a better identification scheme. In [6,9], the authors propose a hierarchical voting algorithm to detect the environment and give the degree of membership to each condition (LOS or NLOS). 

The filtering algorithm is also the basic of a tracking system. Extended Kalman filter (EKF) makes a Taylor expansion of state and measurement equations and keeps the first-order terms to represent the non-linear equations. Only when state and measurement equations are close to linear can the EKF measurement results be close to the true value. UKF can approximate the posterior probability density distribution through the sampling strategy using unscented transformation (UT) [26]. However, the UKF has better performance only when the measurement equation has the characteristic of Gaussian or weak non-Gaussian. When the measurement model is strong non-Gaussian, the accuracy of tracking would drop down. The particle filter can effectively tackle this problem [27]. The core idea of PF is to estimate the probability density function (PDF) of states using random samples in the state space but it will bring about the problem of particle degradation.

Based on the filtering algorithms, NLOS identification algorithms and NLOS mitigation algorithms, many multiple filtering hybrid algorithm have been proposed in [28,29,30]. The IMM [28] combines the soft NLOS identification method with multiple filtering algorithm. According to the transition probability, the measurements are converted into two values: the full LOS and the full NLOS. The two values are filtered separately based on EKF, and then the filtered values are weighted according to the probability given by the likelihood function. The likelihood function gives the probability in a certain environment, so it can be regarded as a soft decision method. However, the IMM algorithm needs priori knowledge of NLOS errors. In order to solve this problem, a robust EKF-based interacting multiple model (R-IMM) hybrid method is investigated in [29], which adds a robust algorithm to locate the target. Due to the application of robust regression algorithm and M-estimation, this algorithm has good robustness under any prior information assumption of NLOS errors. In [30], a tracking and geolocation algorithm based on a semi-parametric approach is proposed, which is also not supported by NLOS priori statistics.

Grey theory is an applied mathematics subject whose research information is partially clear, partially unclear and with uncertain phenomena. Grey prediction is a predicting method for the system containing uncertain factors. It processes the original sequence and obtains the generated sequence. Then the corresponding differential equation is established to predict the future development of things. KF can only be applied in the moving target whose velocity and acceleration is constant and needs to assume the noise characteristic. In [31], the authors firstly apply the grey prediction theory to the vehicle tracking. It can tap the latent laws of target movement and forecast the position of vehicle continuously. But the authors applied this algorithm in video image field instead of WSN. The target tracking algorithm based on the grey innovation model is also proposed in [32]. This method is similar to [31], which directly predicts the next step using the grey prediction model. These methods do not need to make any assumption about the driving noise so they can be adapted to systems affected by random driving noise. However, the localization accuracy is not significantly improved than KF. Furthermore, in [33], the authors combined the grey prediction model with KF. This method continuously updates the state transition matrix based on the grey model and uses this updated matrix to work in the framework of KF. This method could significantly mitigate the driving noise because the state equation is updated according to the motion of mobile nodes.

## 4. Background

### 4.1. Signal Model

Some general concepts in tracking theory are prepared in this section. *M* beacon nodes are randomly deployed in the monitoring area. We use $(x_{i},y_{i}),\;i=1,\ldots ,M$ to denote the coordinate of the beacon nodes. The coordinate of the mobile node at time *k* is (x(k),y(k)), k=1,…,N. The real distance between the *i*-th beacon node and the mobile node at time step *k* can be shown as:

(1) $$d_{i}(k)=\sqrt{{\left(x_{i}-x(k)\right)}^{2}+{\left(y_{i}-y(k)\right)}^{2}}$$

In order to model the measured distance in LOS environment, we add the sensor noise to the real distance:

(2) $$d_{i}^{LOS}(k)=d_{i}(k)+\gamma_{i}$$

where $\gamma_{i}$ is a white Gaussian sensor noise with zero mean and variance $\sigma_{i}^{2}$, this is mainly because numerous factors would affect the measurement value (e.g., the humidity of signal transmission channel, the electronic thermal noise). But each of these interference factors plays minor roles in the final measurement values. According to the central limit theorem, the measurement should obey the Gaussian distribution. So the PDF is described as:

(3) $$f(\gamma_{i})=\frac{1}{\sqrt{2\pi \sigma_{i}^{2}}}\exp\left(-\frac{\gamma_{i}^{2}}{2\sigma_{i}^{2}}\right)$$

In NLOS propagation environment, the signal is always blocked by the obstacle, which suffer the effect of reflection, refraction, scattering and diffraction. So the propagation length is longer than the true distance [4] and we model the measured distance of the *i*-th beacon node at time step *k* as:

(4) $$d_{i}^{NLOS}(k)=d_{i}(k)+\gamma_{i}+\gamma_{NLOS}$$

where $\gamma_{NLOS}$ is the positive NLOS error obeying various distributions (e.g., Gaussian, uniform and exponential). We assume the NLOS error is independent on the sensor noise and obeys different NLOS PDF in different environments as Equations (5)–(7).

The NLOS PDF of $\gamma_{NLOS}$ can be expressed as $\gamma_{NLOS}\sim N(\mu_{NLOS},\;\sigma_{NLOS}^{2})$ under Gaussian distribution with a positive mean.

(5) $$f(\gamma_{NLOS})=\frac{1}{\sqrt{2\pi \sigma_{NLOS}^{2}}}\exp\left(-\frac{{\left(\gamma_{NLOS}^{}-\mu_{NLOS}\right)}^{2}}{2\sigma_{NLOS}^{2}}\right)$$

where $\mu_{NLOS}$ and $\sigma_{NLOS}^{2}$ denote the mean and variance of Gaussian distribution, respectively.

The NLOS PDF of $\gamma_{NLOS}$ can be expressed as $\gamma_{NLOS}\sim U(u_{\min},\;u_{\max})$ under uniform distribution.

(6) $$f(\gamma_{NLOS})=\{\begin{array}{cc} \frac{1}{u_{\max}-u_{\min}} & ,u_{\min}\leq \gamma_{NLOS}\leq u_{\max}\\ 0 & ,else \end{array}$$

where $u_{\min}$ and $u_{\max}$ are the minimum and maximum value of uniform distribution, respectively.

The NLOS PDF of $\gamma_{NLOS}$ can be expressed as $\gamma_{NLOS}\sim E(\lambda )$ under exponential distribution.

(7) $$f(\gamma_{NLOS})=\{\begin{array}{cc} \lambda^{-1}e^{-\gamma_{NLOS}/\lambda } & ,\gamma_{NLOS}\geq 0\\ 0 & ,\gamma_{NLOS}<0 \end{array}$$

where λ is the parameter of Poisson distribution.

### 4.2. A Brief Introduction to the Gaussian Mixture Model (GMM)

Due to the complex and dynamic indoor environment, the distribution of NLOS error is uncertain at every time step. It is difficult to find a real-time analytical density function depicting the NLOS error, but the distribution of NLOS error over a period of time can be clustered roughly through GMM. The core idea of GMM is as follows. According to the central limit theorem from probability theory, any kind of probability density distribution can be approximated by a linear combination of multiple Gaussian density functions, which contain three parameters, that is, mean, variance and weight. The NLOS PDF can be approximated by adjusting these three unknown parameters. We can obtain these unknown parameters through establishing a likelihood function and maximizing it. The likelihood function is established by taking the logarithm of a linear combination of mixed Gaussian functions. Unfortunately, the likelihood function is a non-linear function and direct maximization is not possible. So we employ the expectation-maximization (EM) algorithm to find the maximum likelihood estimation of the unknown parameters. In this paper, the input of GMM is the historical residuals and the current residual, which is the difference between the filtered distance of GKF and the measured distance. The output of GMM is the mean value of each Gaussian distribution.

## 5. Proposed Method

**Figure 1:**
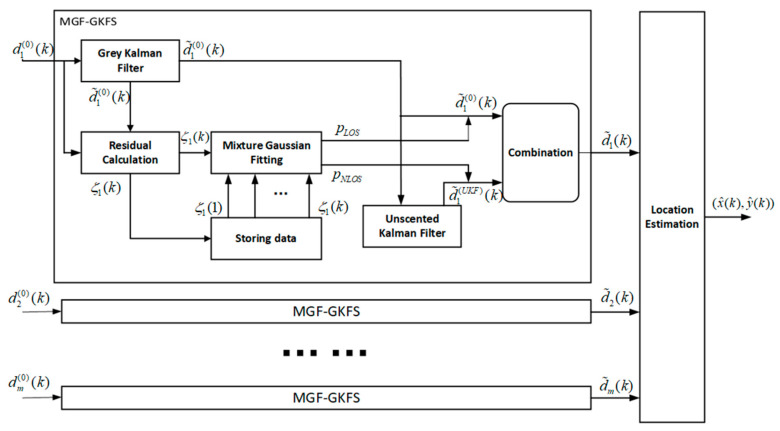
The flowchart of the proposed method.

The flowchart of our algorithm is depicted as Figure 1. The distance value $d_{i}^{\left(0\right)}(k)$ is measured by the sensor nodes and the position estimation $\left(\hat{x}(k),\hat{y}(k)\right)$ is calculated through this filter. Firstly, in order to mitigate the driving noise and initially correct NLOS errors, we utilize GKF to pre-process the measurement. Secondly, we calculate the residual value, which is the difference between the filtered result and the measurement, and save it in the storage module. Thirdly, the historical residuals over a period of time and current residual are utilized to train GMM. Double Gaussian functions are used in this paper to fit these residuals and two clustering centers can be obtained. The degree of membership (probability of propagation condition) is easily obtained through calculating the Euclidean distance from the current residual to the cluster centers. Fourthly, UKF with a relatively large covariance matrix is used to further process the weak NLOS errors after the strong NLOS errors are partially eliminated by GKF. Finally, the filtering results of multiple filters (GKF and UKF) are mixed according to the degree of membership (probability of propagation condition) calculated in a mixture Gaussian fitting step. The maximization likelihood algorithm (ML) is used to estimate the coordinate.

### 5.1. General Concept

#### 5.1.1. The State Equation

In our model, the state vector is a two dimensional vector consisting of the measured distance and velocity, which is denoted as $x_{i}(k)={\left[d_{i}^{\left(0\right)}(k),\;\;{\dot{d}}_{i}^{\left(0\right)}(k)\right]}^{T}\;,i=1,\ldots ,M,\;\;k=1,\ldots ,N$. The movement of the mobile node can be described as follows:

(8) $$x_{i}(k)=Fx_{i}(k-1)+C\nu_{i}(k-1)$$

where $F=\left[\begin{array}{cc} 1 & \Delta t\\ 0 & 1 \end{array}\right]$ is the state transition matrix, $C=\left[\begin{array}{c} \Delta t^{2}/2\\ \Delta t \end{array}\right]$, $\Delta t=t_{k}-t_{k-1}$ is the time interval of sampling. Driving noise $\nu_{i}(k)$ is caused by the random acceleration of the target. The PDF of driving noise is under white Gaussian distribution whose covariance matrix is $Q_{i}(k)=\sigma_{\nu ,i}^{2}I_{2}$.

#### 5.1.2. The Measurement Equation

Measurement equation of i-th beacon node at time step k is expressed as follows:

(9) $$z_{i}(k)=Hx_{i}(k-1)+w_{i}(k)$$

where the observation matrix H=[1,0]. In the actual tracking environment, the measurement noise consists of sensor noise and NLOS error, whose covariance matrix can be denoted as $J(k)=diag([\sigma_{1}^{2},\sigma_{2}^{2},\ldots ,\sigma_{m}^{2}]),m=1,\ldots ,M$, where the $\sigma_{m}^{2}$ is expressed as follows:

(10) $$\sigma_{m}^{2}=\{\begin{array}{ll} \sigma_{i}^{2} & ,LOS\\ \sigma_{i}^{2}+\sigma_{NLOS}^{2} & ,NLOS \end{array}$$

We assume the sensor noise $\sigma_{i}^{2}$ is already known while $\sigma_{NLOS}^{2}$ is unknown. The value of $\sigma_{i}^{2}$ in the filter should be initiated according to the size of scenario or sensing radius.

### 5.2. Grey Kalman Filter (GKF)

GKF is based on the KF framework. The difference from KF is that GM(1,1) is used in the prediction step, where, ‘G’ means grey, ‘M’ means model and ‘(1,1)’ denotes the established differential equation is first-order and has only one variable. The generation value of GM (1,1) is directly used as the predicted value of the next time step in KF. Furthermore, the state transition matrix is updated continuously based on the motion characteristic of targets. The new state transition matrix is applied to the updating process of the covariance matrix and state estimation at the current time step. Therefore, GKF could avoid the random direction and speed of the targets which cause the process noise in the state equation. Due to the prediction step being dependent on the historical filtered values, GKF could also mitigate NLOS errors partially.

The entire algorithm is elaborated as following steps:

Step 1: Building the original distance series.

We define the *n*-th element of the original series of *i*-th beacon node at time step *k* as follows:

(11) $$y_{i,k}^{\left(0\right)}(n)={\tilde{d}}_{i}^{\left(0\right)}(k-l_{c}+n),i=1,\ldots ,M,k=l_{c},\ldots ,N,n=1,\ldots ,l_{c}$$

Initial the original time series as follows:

(12) $$\begin{array}{ll} y_{i,k}^{\left(0\right)} & =(y_{i,k}^{\left(0\right)}(1),y_{i,k}^{\left(0\right)}(2),\ldots y_{i,k}^{\left(0\right)}(l_{c}))\\ & =({\tilde{d}}_{i}^{\left(0\right)}(k-l_{c}+1),{\tilde{d}}_{i}^{\left(0\right)}(k-l_{c}+2),\ldots ,{\tilde{d}}_{i}^{\left(0\right)}(k)),k=l_{c},\ldots ,N,n=1,\ldots ,l_{c} \end{array}$$

where $y_{i,k}^{\left(0\right)}$ is the original distance series, ${\tilde{d}}_{i}^{\left(0\right)}(k)$ denotes the filtered distance at time step *k*, $l_{c}$ is the constant series length of predicting points. The predicting points are the historical GKF filtered distances and we can choose different series length applying to prediction step.

For example, if we set $l_{c}=3$, we obtain original distance series as follows:

(13) $$y_{i,k}^{\left(0\right)}=({\tilde{d}}_{i}^{\left(0\right)}(k-2),{\tilde{d}}_{i}^{\left(0\right)}(k-1),{\tilde{d}}_{i}^{\left(0\right)}(k)),i=1,\ldots ,M,k=l_{c},\ldots ,N$$

where the ${\tilde{d}}_{i}^{\left(0\right)}(k-2),{\tilde{d}}_{i}^{\left(0\right)}(k-1),{\tilde{d}}_{i}^{\left(0\right)}(k)$ are the prediction points. We would use these points to predict the value of the next time step. The number of prediction points must be chosen reasonably. Too many prediction points will bring in lots of historical values that are too far for the present moment so that the premature value can be regarded as noise. However, too few prediction points also cannot be accepted because they fail to provide enough information for prediction [33]. The paper [33] has verified that the appropriate number of prediction points is about 8 when the sampling period is 1 s. In actual projects, engineers need to set a reasonable prediction points number according to the sampling frequency. In the experimental part of this paper, the sampling interval is 1 s so 8 prediction points are set. At the first 8 points, because the prediction points are too few for grey prediction ($k=l_{c},\ldots ,N$), KF is used to initial the time series. Thereafter, the number of historical values is enough for adopting GKF to filter the measured distance.

Step 2: Calculate the first-order accumulative series as Equation (15) through adding all the past prediction points for every element in the series $y_{i,k}^{\left(0\right)}$.

We define the element of accumulative series as Equation (14):

(14) $$y_{i,k}^{\left(1\right)}(n)=\{\begin{array}{c} {\tilde{d}}_{i}^{\left(0\right)}(k-l_{c}+1),n=1\\ \sum_{j=k-l_{c}+1}^{k-l_{c}+n}{\tilde{d}}_{i}^{\left(0\right)}(j),n>1 \end{array}$$

Calculate the first-order accumulative series as follows:

(15) $$\begin{array}{cc} y_{i,k}^{\left(1\right)} & =(y_{i,k}^{\left(1\right)}(1),y_{i,k}^{\left(1\right)}(2),\ldots ,y_{i,k}^{\left(1\right)}(n),\ldots y_{i,k}^{\left(1\right)}(l_{c}))\\ & =({\tilde{d}}_{i}^{\left(0\right)}(k-l_{c}+1),\sum_{j=k-l_{c}+1}^{k-l_{c}+2}{\tilde{d}}_{i}^{\left(0\right)}(j),\ldots ,\sum_{j=k-l_{c}+1}^{k-l_{c}+n}{\tilde{d}}_{i}^{\left(0\right)}(j),\ldots \sum_{j=k-l_{c}+1}^{k}{\tilde{d}}_{i}^{\left(0\right)}(j)),i=1,\ldots M,k=l_{c},\ldots ,N \end{array}$$

where $y_{i,k}^{\left(1\right)}$ denotes the accumulative series that will be utilized to predict the distance value at the next time step.

Step 3: Assume that the *n*-th element $y_{i,k}^{\left(1\right)}(n)$ in series $y_{i,k}^{\left(1\right)}$ meets the first-order ordinary differential equation as (16).

(16) $$\frac{dy_{i,k}^{\left(1\right)}(n)}{dn}+a_{i,k}y_{i,k}^{\left(1\right)}=u_{i,k},i=1,\ldots ,M,k=l_{c},\ldots ,N$$

where $a_{i.k}$ is development coefficient, $u_{i.k}$ is grey effect coefficient in the grey theory. As long as $a_{i,k}$ and $u_{i,k}$ are obtained, the predicted value ${\hat{y}}_{i,k}^{\left(1\right)}(n+1)$ can be solved.

Step 4: Calculate the mean of the accumulated series between every two prediction points to obtain matrix B(k) and generate the constant term vector c(k) as follows.

(17) $$B(k)=\left[\begin{array}{cc} -0.5(y_{i,k}^{\left(1\right)}(1)+y_{i,k}^{\left(1\right)}(2)) & 1\\ -0.5(y_{i,k}^{\left(1\right)}(2)+y_{i,k}^{\left(1\right)}(3)) & 1\\ \ldots & \ldots \\ -0.5(y_{i,k}^{\left(1\right)}(n-1)+y_{i,k}^{\left(1\right)}(n)) & 1 \end{array}\right],\;c(k)=\left[\begin{array}{c} {\tilde{d}}_{i}^{\left(0\right)}(k-l_{c}+2)\\ {\tilde{d}}_{i}^{\left(0\right)}(k-l_{c}+3)\\ \ldots \\ {\tilde{d}}_{i}^{\left(0\right)}(k) \end{array}\right],\;n=1,\ldots ,l_{c},\;k=l_{c},\ldots ,N$$

Step 5: Calculate $a_{i,k}$ and $u_{i,k}$ through the least-square method.

(18) $$\left[\begin{array}{c} a_{i,k}\\ u_{i,k} \end{array}\right]={\left(B{\left(k\right)}^{T}B(k)\right)}^{-1}B{\left(k\right)}^{T}c(k),i=1,\ldots ,M,k=l_{c},\ldots ,N$$

Step 6: Substitute $a_{i,k}$ and $u_{i,k}$ to the differential Equation (16) and calculate the solution ${\hat{y}}_{i,k}^{\left(1\right)}(n)$.

(19) $${\hat{y}}_{i,k}^{\left(1\right)}(n)=({\tilde{d}}_{i}^{\left(0\right)}(k-l_{c}+1)-\frac{u_{i,k}}{a_{i,k}})e^{-a_{i}n}+\frac{u_{i,k}}{a_{i,k}},k=l_{c},\ldots ,N,n=1,\ldots ,l_{c}$$

We can use this model to estimate ${\hat{y}}_{i,k}^{\left(1\right)}(n+1)$ by substituting n+1 to Equation (19) and obtaining:

(20) $${\hat{y}}_{i,k}^{\left(1\right)}(n+1)=({\tilde{d}}_{i}^{\left(0\right)}(k-l_{c}+1)-\frac{u_{i,k}}{a_{i,k}})e^{-a_{i}(n+1)}+\frac{u_{i,k}}{a_{i,k}},k=l_{c},\ldots ,N,n=1,\ldots ,l_{c}$$

Step 7: Build the predicted series ${\hat{y}}_{i,k}^{\left(0\right)}$ as (22) through cumulative subtraction of the above results as Equation (21) and the prediction of the next time step ${\hat{d}}_{i}^{\left(0\right)}(k+1)$ can be calculated as Equation (23).

(21) $$\{\begin{array}{l} {\hat{y}}_{i,k}^{\left(0\right)}(n)={\hat{y}}_{i,k}^{\left(1\right)}(n)-{\hat{y}}_{i,k}^{\left(1\right)}(n-1),n>1\\ {\hat{y}}_{i,k}^{\left(0\right)}(1)={\tilde{d}}_{i}^{\left(0\right)}(k-l_{c}+1),n=1 \end{array}$$

(22) $$\begin{array}{ll} {\hat{y}}_{i,k}^{\left(0\right)} & =({\hat{y}}_{i,k}^{\left(0\right)}(1),{\hat{y}}_{i,k}^{\left(0\right)}(2),\ldots ,{\hat{y}}_{i,k}^{\left(0\right)}(n))\\ & =({\tilde{d}}_{i}^{\left(0\right)}(k-l_{c}+1),{\hat{d}}_{i}^{\left(0\right)}(k-l_{c}+2),\ldots ,{\hat{d}}_{i}^{\left(0\right)}(k-l_{c}+n)),n=1,\ldots ,l_{c},k=l_{c},\ldots ,N \end{array}$$

(23) $${\hat{d}}_{i}^{\left(0\right)}(k+1)={\hat{y}}_{i,k}^{\left(1\right)}(n+1)-{\hat{y}}_{i,k}^{\left(1\right)}(n),n=1,\ldots ,l_{c},k=l_{c},\ldots ,N$$

${\hat{d}}_{i}^{\left(0\right)}(k+1)$ is directly used as the value of the next time in the prediction step in KF. It plays a significant role in eliminating NLOS errors. In the case of NLOS, the measured distance is much longer than the real distance. Therefore, in the continuous time series of distance, severe NLOS errors will cause the mutation point. But the predicted value of GM (1,1) is highly correlated with previous filtered values, and large fluctuations will be weakened, which can effectively solve this issue.

In order to simplify the expression, set parameter α and β and rewrite the predicted value as Equations (23) and (24) [31].

(24) $$\alpha_{i,k}=\frac{a_{i,k}}{1+0.5a_{i,k}},\beta_{i,k}=\frac{u_{i,k}}{1+0.5a_{i,k}}$$

(25) $${\hat{d}}_{i}^{\left(0\right)}(k+1)=(\beta_{i,k}-\alpha_{i,k}{\tilde{d}}_{i}^{\left(0\right)}(k-l_{c}+1))\exp(-a_{i,k}(k-2))$$

Similarly, for speed series we can also get its prediction as Equation (26) using the same method of distance prediction.

(26) $${\hat{v}}_{i,k}^{\left(1\right)}(n+1)=({\tilde{v}}_{i}^{\left(0\right)}(k-l_{c}+1)-\frac{{\overset{⌢}{u}}_{i,k}}{{\overset{⌢}{a}}_{i,k}})e^{-{\overset{⌢}{a}}_{i}(n+1)}+\frac{{\overset{⌢}{u}}_{i,k}}{{\overset{⌢}{a}}_{i,k}},n=1,\ldots ,l_{c},k=l_{c},\ldots ,N$$

where $v_{i,k}^{\left(1\right)}$ is the accumulative series value of speed. ${\overset{⌢}{u}}_{i,k}$ and ${\overset{⌢}{a}}_{i,k}$ are used to distinguish the parameter $a_{i,k}$ and $u_{i,k}$ in distance prediction.

Step 8: Obtain the predicted state at the next time step.

(27) $${\hat{x}}_{i}(k|k-1)={\left[{\hat{d}}_{i}^{\left(0\right)}(k+1),{\hat{v}}_{i}^{\left(0\right)}(k+1)\right]}^{T}$$

where ${\hat{d}}_{i}^{\left(0\right)}(k+1)$ is the distance predicted in (25) and ${\hat{v}}_{i}^{\left(0\right)}(k+1)$ can similarly be estimated.

Step 9: Update the state transition matrix ${\hat{F}}_{i}$ as Equation (28) [33].

(28) $${\hat{F}}_{i}=\left[\begin{array}{cc} e^{-a_{i,k}} & 0\\ 0 & e^{-{\overset{⌢}{a}}_{i,k}} \end{array}\right]$$

This step has an important effect on the mitigation of process noise caused by random direction and speed, since the state transition matrix is updated at every time step according to the target movement characteristics.

Step 10: Kalman Filter.

Covariance prediction:

(29) $$P_{i}(k|k-1)={\hat{F}}_{i}P_{i}(k-1|k-1){\hat{F}}_{i}{}^{T}+Q_{i}(k-1)$$

Calculate measurement residual $e_{i}(k)$ and Kalman gain $K_{i}(k)$:

(30) $$e_{i}(k)=z_{i}(k)-Hx_{i}(k|k-1)$$

(31) $$K_{i}(k)=P_{i}(k|k-1)H^{T}{\left[HP_{i}(k|k-1)H^{T}+R_{i}(k)\right]}^{-1}$$

Obtain the position estimation through estimation Equations (32) and (33).

(32) $$x_{i}(k|k)=x_{i}(k|k-1)+K_{i}(k)e_{i}(k)$$

(33) $$P_{i}(k|k)=\left[I_{2}-K_{i}(k)H\right]P_{i}(k|k-1)$$

In order to show our algorithm clearly, we give the pseudo code in this paper as Algorithm 1.
**Algorithm 1.** The pre-processing for the measurements based on grey Kalman filter (GKF) **Input:**${\tilde{d}}_{i}^{\left(0\right)}(k-l_{c}+1),\ldots ,{\tilde{d}}_{i}^{\left(0\right)}(k)$**Output:**${\hat{d}}_{i}^{\left(0\right)}(k+1)$**begin****for***i* = 1: *M*
**do**  **for**
$k=l_{c}:N$
**do**
   $sum(k-l_{c}+1)={\tilde{d}}_{i}^{\left(0\right)}(k-l_{c}+1)$   c=[]      B=[  ]    **for**
*j*=$k-l_{c}+2:k$
**do**     $sum(j)=sum(j-1)+{\tilde{d}}_{i}^{\left(0\right)}(j)$     B=[B;−0.5∗sum(j)−0.5∗sum(j−1),1]     $c=\left[c;{\tilde{d}}_{i}^{\left(0\right)}(j)\right]$
    **end for**
   ${\left[a,u\right]}^{T}={\left(B\prime B\right)}^{-1}B\prime c$   $\begin{array}{l} alpha=a/(1+0.5a)\;\\ beta=u/(1+0.5a) \end{array}$   ${\hat{d}}_{i}^{\left(0\right)}(k+1)=(beta-alpha*{\tilde{d}}_{i}^{\left(0\right)}(k-l_{c}+1))\exp(-alpha*(k-2)))$   $\hat{F}=\left[\begin{array}{llll} \exp(-a) & 0; & 0 & \exp(-\overset{⌢}{a}) \end{array}\right]$    $P_{i}(k|k-1)=\hat{F}P_{i}(k-1|k-1)\hat{F}\prime +\sigma_{LOS}^{2}CC\prime$
   $e_{i}(k)=z_{i}(k)-Hx_{i}(k|k-1)$    $K_{i}(k)=P_{i}(k|k-1)H^{T}inv(HP_{i}(k|k-1)H^{T}+R_{i}(k))$    $x_{i}(k|k)=x_{i}(k|k-1)+K_{i}(k)e_{i}(k)$    $P_{i}(k|k)=(eye(2)-K_{i}(k)H)P_{i}(k|k-1)$
  **end for**  **for**
$k=1:l_{c}$
**do**
   $x_{i}(k|k-1)=Fx_{i}(k-1|k-1)$
   $P_{i}(k|k-1)=FP(k-1|k-1)F\prime +\sigma_{LOS}^{2}CC\prime$
   $e_{i}(k)=z_{i}(k)-Hx_{i}(k|k-1)$
   $K_{i}(k)=P_{i}(k|k-1)H^{T}inv(HP_{i}(k|k-1)H^{T}+R_{i}(k))$
   $x_{i}(k|k)=x_{i}(k|k-1)+K_{i}(k)e_{i}(k)$   $P_{i}(k|k)=(eye(2)-K_{i}(k)H)P_{i}(k|k-1)$
  **end for** **end for****end**

**Figure 2:**
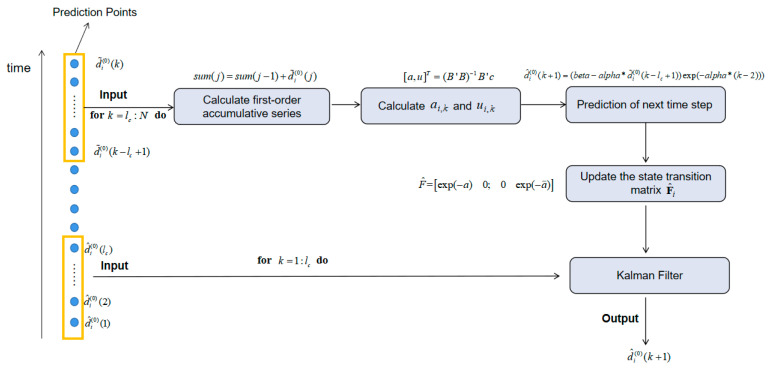
The flowchart of GKF.

In order to follow the pseudocode in Algorithm 1 and provide a much clearer exposition of GKF, a more detailed flowchart (Figure 2) is prepared corresponding to the GKF of Figure 1.

### 5.3. Mixture Gaussian Fitting (MGF)

A soft NLOS identification method is proposed based on GMM clustering algorithm. By contrast with from the hard decision method such as Wylie, Rwgh [4] and the hypothesis testing discriminant method [11], our method give the probability of each condition, which we regard this method as a soft NLOS identification way.

The effectiveness of mixture Gaussian fitting (MGF) is dependent on the corrected distance in pre-processing step. GKF can mitigate the process noise and alleviate NLOS errors partially. Therefore, the filtered distance of GKF can be used to calculate the residual which is the difference from the measurement. So the residual value here is essentially a NLOS error estimated. In general, it is impractical to obtain the NLOS error distribution parameters, but in this paper, GMM is used to approximate the PDF of NLOS errors and gives the probability of each condition.

**Step 1 (Calculate the residual):**

We firstly calculate the distance residual between the measured value and the filtered value of GKF and store it to the memory module.

(34) $$\zeta_{i}(k)=|{\tilde{d}}_{i}^{\left(0\right)}(k)-d_{i}^{\left(0\right)}(k)|,i=1,\ldots ,M,k=l_{c},\ldots ,N$$

**Step 2 (Fit the mixture Gaussian distribution):**

We use the historical residuals and the current residual to fit the mixture Gaussian distribution. The distribution of mixture Gaussian is as follows:

(35) $$T_{i}(\theta )=\sum_{j=1}^{G}\frac{w_{j}}{\sqrt{2\pi \sigma_{j}^{2}}}\exp(-\frac{1}{2}\frac{{\left(\zeta_{i}(k)-\mu_{j}\right)}^{2}}{\sigma_{j}^{2}})$$

where $\theta =(\theta_{1},\theta_{2},\ldots ,\theta_{G})$ is the unknown Gaussian parameter vector, the distribution parameter of each distribution $\theta_{j}=(\mu_{j},\sigma_{j},w_{j})$. $\mu_{j}$ and $\sigma_{j}^{}$ are the mean and the standard variance, $\mu_{1}<\mu_{2}<\ldots <\mu_{G}$, $w_{j}$ is the probability of the *j*-th Gaussian function. In this paper, two Gaussian functions are used to fit these residuals for every beacon node. So *G* is set to 2. One has a relatively small mean value, which can represent the LOS condition or the weak NLOS condition, the other has a relatively large mean value which indicates the strong NLOS condition.

Take the logarithm to (35) to obtain the likelihood function $L_{i}^{k}(\theta )$. [11]

(36) $$L_{i}^{k}(\theta )=\sum_{n=1}^{k}\log\sum_{j=1}^{G}\frac{w_{j}}{\sqrt{2\pi \sigma_{j}^{2}}}\exp(-\frac{1}{2}\frac{{\left(\zeta_{i}(n)-\mu_{j}\right)}^{2}}{\sigma_{j}^{2}})$$

We use expectation maximization (EM) algorithm [11] to estimate the unknown parameter θ through maximizing the log-likelihood function $L_{i}^{k}(\theta )$. The EM algorithm continuously iterates and adjusts the parameter θ to achieve the maximum value of the likelihood function. In practice, a termination condition is usually set as Equation (37).

(37) $$L_{i}^{k}(\theta^{r+1})-L_{i}^{k}(\theta^{r})\leq \varepsilon$$

where the parameter ε is a small threshold which is 1 × 10^−5^ in this paper and the parameter *r* is the iteration times. The basic process of the EM algorithm consists of E-step and M-step.

- E-step. Calculate the probability that the residual value $\zeta_{i}(k)$ belongs to the *j*-th Gaussian distribution as Equation (38).
  (38) $$Y_{j}^{t}(\zeta_{i}(k))=\frac{w_{j}N(\zeta_{i}(k)|\mu_{j},\sigma_{j}^{2})}{\sum_{j=1}^{G}w_{j}N(\zeta_{i}(k)|\mu_{j},\sigma_{j}^{2})},j=1,2,\ldots ,G$$
- M-step. Calculate the partial derivative of the Equation (36) and let the derivative function equal to zero. Solve unknown parameters of Gaussian distribution as Equations (39)–(41).
  (39) $$\mu_{j,i}^{t}(k)=\sum_{j=1}^{G}Y_{j}^{t}({\tilde{d}}_{i}^{\left(0\right)}(k)){\tilde{d}}_{i}^{\left(0\right)}(k)/\sum_{j=1}^{G}Y_{j}^{t}({\tilde{d}}_{i}^{\left(0\right)}(k)),i=1,\ldots ,M$$
  (40) $$\sigma_{j,i}^{t}(k)=\sum_{j=1}^{G}Y_{j}^{t}{\left({\tilde{d}}_{i}^{\left(0\right)}(k)-\mu_{j,i}^{t}(k)\right)}^{2}/\sum_{j=1}^{G}Y_{j}^{t}({\tilde{d}}_{i}^{\left(0\right)}(k)),i=1,\ldots ,M$$
  (41) $$w_{j,i}^{t}(k)=\frac{1}{G}Y_{j}^{t}({\tilde{d}}_{i}^{\left(0\right)}(k)),i=1,\ldots ,M$$

**Step 3 (Calculate the degree of membership in each condition)**

After using two Gaussian distribution functions to fit all the residual value, we utilize the Euclidean distance between current residual and the clustering centre $\mu_{j,i}(k)$ to calculate the degree of membership in each condition. We define the membership function as (44) and (45):

(42) $$dis_{LOS}^{i}(k)=\left|\zeta_{i}(k)-\mu_{1,i}(k)\right|$$

(43) $$dis_{NLOS}^{i}(k)=\left|\zeta_{i}(k)-\mu_{2,i}(k)\right|$$

(44) $$p_{LOS}^{i}(k)=\frac{dis_{NLOS}^{i}(k)}{dis_{LOS}^{i}(k)+dis_{NLOS}^{i}(k)}$$

(45) $$p_{NLOS}^{i}(k)=\frac{dis_{LOS}^{i}(k)}{dis_{LOS}^{i}(k)+dis_{NLOS}^{i}(k)}$$

**Figure 3:**
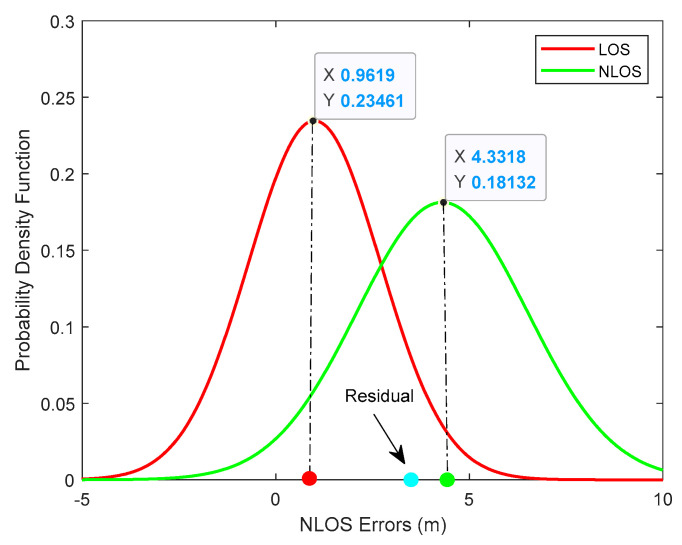
The mixture Gaussian fit result.

To illustrate the meaning of the membership function clearly, we will provide an example when NLOS errors obey $N(5,4^{2})$ and we choose the fitting result at one time step as Figure 3.

We can observe that the residual value at this time step is 3.3 m (blue dot) and the means of Gaussian functions are 1.0 m (red dot) and 4.3 m (green dot) respectively. We have successfully fitted the NLOS errors distribution with two Gaussian functions through the EM algorithm. It can be observed that the fitted result is approximately in line with the real condition. The sensor noise obeys zero-mean white Gaussian distribution which is very close to the red curve while the NLOS errors obey $N(5,4^{2})$ which is approximately with consistent with the green curve. The Euclidean distance between the residual and the red dot is (3.3 − 1.0) m = 2.3 m. Similarly, the Euclidean distance between the residual and the green dot is (4.3 − 3.3) m = 1.0 m. It is not difficult to understand that the farther away from the mean of a specific Gaussian distribution, the lower the probability that it belongs to this Gaussian distribution. In this example, the membership of the residual value to the red Gaussian distribution is 1.0/(2.3 + 1.0) m = 0.3 and the membership of green Gaussian is 0.7. In order to display our identification algorithm clearly we give the description of MGF as Algorithm 2.
**Algorithm 2.** Pseudocode of mixture Gaussian fitting (MGF) **Input:**${\tilde{d}}_{i}^{\left(0\right)}(k),d_{i}^{\left(0\right)}(k),state\_num$**Output:**$p_{LOS}^{i}(k),p_{NLOS}^{i}(k)$**begin****for**i=1:M**do**  **for**
k=1:N
**do**
   $res(i,k)=abs({\tilde{d}}_{i}^{\left(0\right)}(k)-d_{i}^{\left(0\right)}(k))$
   res_save=res(i,1:k)
   [Priors0,Mu0,Sigma0]=EM_init_kmeans(res_save,state_num)
   [Priors,Mu,Sigma]=EM(res_save,Priors0,Mu0,Sigma0)
   sort(Mu)
   dis_los=abs(res(i,k)−Mu(1))
   dis_nlos=abs(res(i,k)−Mu(2))
   $p_{LOS}^{i}(k)=dis\_nlos/(dis\_los+dis\_nlos)$
   $p_{NLOS}^{i}(k)=dis\_los/(dis\_los+dis\_nlos)$
  **end for****end for****end**

### 5.4. Unscented Kalman Filter (UKF)

To simplify the subsequent analysis, we rewrite the system Equations (8) and (9) using symbol f(·) and h(·):

(46) $$x_{i}(k+1)=f\left(x_{i}(k),v_{i}(k)\right)$$

(47) $$z_{i}(k)=h\left(x_{i}(k),w_{i}(k)\right)$$

**Step 1 (Initialization)**

Calculate the expectation of the processed state and the covariance by GKF.

(48) $${\bar{x}}_{i}=E({\tilde{x}}_{i})$$

(49) $$P_{i}=E\left[({\tilde{x}}_{i}-{\bar{x}}_{i}){\left({\tilde{x}}_{i}-{\bar{x}}_{i}\right)}^{T}\right]$$

where ${\tilde{x}}_{i}={\left[{\tilde{d}}_{i}^{\left(0\right)}(k),{\tilde{v}}_{i}^{\left(0\right)}(k)\right]}^{T}$ denotes the state vector after being pre-processed by GKF.

**Step 2 (Generate the sigma points)**

The dimension of sigma points is 2*L* + 1 where *L* is the dimension of the state vector [4]. In this paper, *L* = 2. The generated sigma points are expressed as follows:

(50) $${\tilde{x}}_{i}^{\left(j\right)}(k-1)=\{\begin{array}{ll} {\bar{x}}_{i} & j=0\\ {\bar{x}}_{i}+{\left(\sqrt{\left(n+\delta )P_{i}(k-1\right)}\right)}_{j} & j=1,\ldots ,L\\ {\bar{x}}_{i}-{\left(\sqrt{\left(n+\delta )P_{i}(k-1\right)}\right)}_{j} & j=L+1,\ldots ,2L \end{array}$$

where δ is the distribution parameter of UKF.

**Step 3 (Prediction)**

Transform the sigma points as Equation (51) based on the non-linear state Equation (46). And calculate the weighted average of these sigma points as Equation (52).

(51) $${\tilde{x}}_{i}^{\left(j\right)}(k|k-1)=f({\tilde{x}}_{i}^{\left(j\right)}(k-1))$$

(52) $${\hat{x}}_{i}(k|k-1)=\sum_{j=0}^{2L}\omega_{i}^{j}{\tilde{x}}_{i}^{\left(j\right)}(k|k-1)$$

(53) $$P_{i}(k|k-1)=\sum_{j=0}^{2L}\omega_{i}^{j}({\tilde{x}}_{i}^{\left(j\right)}(k|k-1)-{\hat{x}}_{i}(k-1)){\left({\tilde{x}}_{i}^{\left(j\right)}(k|k-1)-{\hat{x}}_{i}(k-1)\right)}^{T}+Q_{\alpha },Q_{\alpha }=2\sigma_{i}^{2}$$

(54) $$\omega_{i}^{j}=\{\begin{array}{cc} 1-\frac{L}{\delta } & j=0\\ \frac{1}{2\delta } & j=1,\ldots ,2L \end{array}$$

where $\omega_{j}$ is the weighting factor.

Thereafter, predict the observation value using nonlinear measurement equation [4].

(55) $$z_{i}^{\left(j\right)}(k|k-1)=h({\tilde{x}}_{i}^{\left(j\right)}(k|k-1))$$

Use the weighting factor to obtain the average value of $z_{i}^{\left(j\right)}(k)$.

(56) $${\hat{z}}_{i}(k|k-1)=\sum_{j=0}^{2L}\omega_{i}^{j}\cdot z_{i}^{\left(j\right)}(k|k-1)$$

**Step 4 (Update)**

Update the matrix of state covariance as Equation (57) and the measurement covariance as Equation (58) [4].

(57) $$P_{i}^{f}(k)=\sum_{j=0}^{2L}\omega_{i}^{j}({\tilde{x}}_{i}^{\left(j\right)}(k|k-1)-{\hat{x}}_{i}(k|k-1)){\left({\tilde{x}}_{i}^{\left(j\right)}(k|k-1)-{\hat{x}}_{i}(k|k-1)\right)}^{T}$$

(58) $$P_{i}^{h}(k)=\sum_{j=0}^{2L}\omega_{i}^{j}\cdot (z_{i}^{\left(j\right)}(k|k-1)-{\hat{z}}_{i}(k|k-1))\cdot {\left(z_{i}^{\left(j\right)}(k|k-1)-{\hat{z}}_{i}(k|k-1)\right)}^{T}+R_{i}$$

Calculate the Kalman gain as Equation (59), the updated state vector as Equation (60) and the covariance matrix as Equation (61).

(59) $$K_{i}(k)=P_{i}^{f}(k)P_{i}^{h}{\left(k\right)}^{-1}$$

(60) $${\hat{x}}_{i}(k)={\hat{x}}_{i}(k|k-1)+K_{i}(k)\cdot (z_{i}(k)-{\hat{z}}_{i}(k|k-1))$$

(61) $$P_{i}(k)=P_{i}(k|k-1)-K_{i}(k)P_{i}^{z}(k)K_{i}^{T}(k)$$

The final filtered distance of UKF is denoted as ${\tilde{d}}_{i}^{\left(UKF\right)}(k)$.

### 5.5. Data Fusion and Position Estimation

Distance filtering results for two filters (GKF and UKF) can be mixed according to the probability in each condition given by MGF. The final distance filtered value is expressed as:

(62) $${\tilde{d}}_{i}(k)={\tilde{d}}_{i}^{\left(0\right)}(k)\cdot p_{LOS}^{i}(k)+{\tilde{d}}_{i}^{\left(UKF\right)}(k)\cdot p_{NLOS}^{i}(k)$$

The coordinates of the mobile and the filtered distances satisfy the following equations [9]:

(63) $$\{\begin{array}{c} {\left(x_{1}-\hat{x}(k)\right)}^{2}+{\left(y_{1}-\hat{y}(k)\right)}^{2}={\left({\tilde{d}}_{1}(k)\right)}^{2}\\ \ldots \\ {\left(x_{m}-\hat{x}(k)\right)}^{2}+{\left(y_{m}-\hat{y}(k)\right)}^{2}={\left({\tilde{d}}_{m}(k)\right)}^{2} \end{array}$$

The equation set is unable to find solution directly. The estimation of position can be calculated by ML algorithm and we rewrite the equation set as AX=b, where:

(64) $$A=2\left[\begin{array}{cc} \left(x_{1}-x_{2}\right) & \left(y_{1}-y_{2}\right)\\ \left(x_{1}-x_{3}\right) & \left(y_{1}-y_{3}\right)\\ \ldots & \ldots \\ \left(x_{1}-x_{M}\right) & \left(y_{1}-y_{M}\right) \end{array}\right],b=\left[\begin{array}{c} {\tilde{d}}_{2}{\left(k\right)}^{2}-{\tilde{d}}_{1}(k)-(x_{2}^{2}+y_{2}^{2})+(x_{1}^{2}+y_{1}^{2})\\ {\tilde{d}}_{3}{\left(k\right)}^{2}-{\tilde{d}}_{1}(k)-(x_{3}^{2}+y_{3}^{2})+(x_{1}^{2}+y_{1}^{2})\\ \ldots \\ {\tilde{d}}_{m}{\left(k\right)}^{2}-{\tilde{d}}_{1}(k)-(x_{m}^{2}+y_{m}^{2})+(x_{1}^{2}+y_{1}^{2}) \end{array}\right]$$

Then the coordinate of mobile nodes can be obtained:

(65) $$\left[\begin{array}{c} {\hat{x}}_{i}(k)\\ {\hat{y}}_{i}(k) \end{array}\right]={\left(A^{T}A\right)}^{-1}A^{T}b$$

## 6. Simulation and Experiment Results

### 6.1. Simulation Results

**Figure 4:**
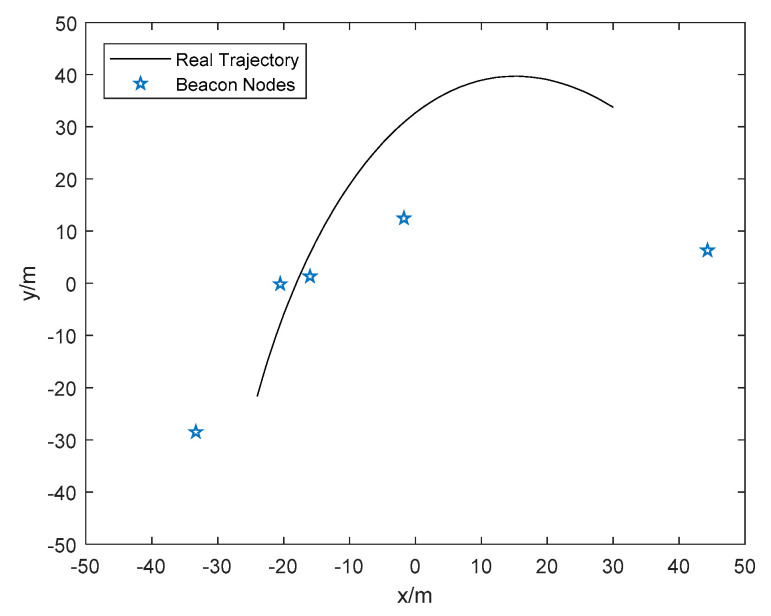
The trajectory and the position of beacon nodes in one Monte Carlo run.

In order to verify the effectiveness of the proposed algorithm in a complex indoor environment, full-scale simulations are conducted. The classic EKF based algorithm IMM [26] and advanced robust extended Kalman filter (REKF)-based algorithm R-IMM [29] are adopted for comparison. Various NLOS distributions with different parameters are adopted to validate the robustness of our method. The mobile node is moving in a square of 100 m × 100 m. For each distribution parameter, the simulation is run 1000 times to obtain the statistical results. The beacon nodes are deployed randomly for each Monte Carlo simulation. The trajectory and the deployment of beacon nodes in one of the Monte Carlo runs are shown in the Figure 4. The mean error distance (MED) is the mean Euclidean distance between the position estimation and the real location, which is chosen to evaluate the localization accuracy in this paper. The MED is defined as follows:

(66) $$MED=\frac{1}{T_{n}N}\sum_{i=1}^{Tn}\sum_{k=1}^{N}\sqrt{{\left({\hat{x}}_{i}(k)-x_{i}(k)\right)}^{2}+{\left({\hat{y}}_{i}(k)-y_{i}(k)\right)}^{2}}$$

where $T_{n}$ is the number of Monte Carlo running, *N* is the total time of the mobile node moving.

The default parameters’ values under different NLOS distributions are shown as Table 2.

#### 6.1.1. Gaussian Distribution

**Figure 5:**
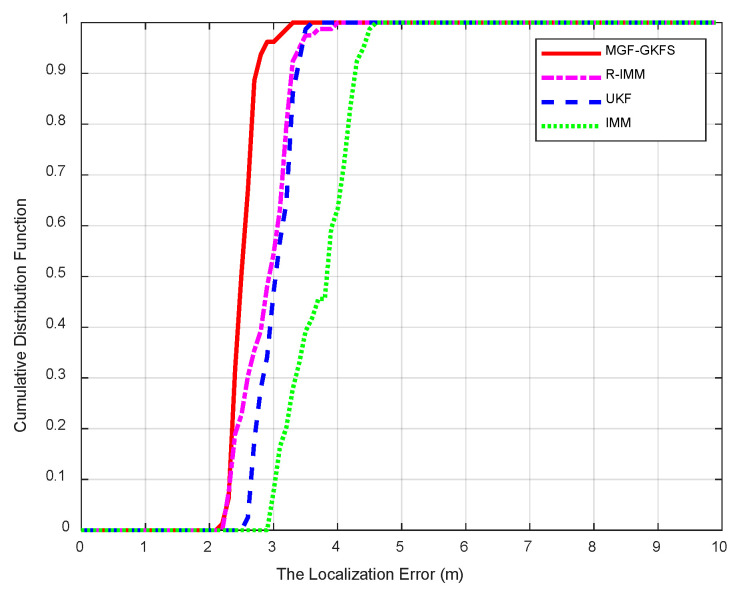
The cumulative distribution function (CDF) when non-line of sight (NLOS) errors obey $N(2,4^{2})$.

The cumulative distribution function (CDF) based on the MED when NLOS errors obey $N(2,4^{2})$ is depicted in Figure 5. The average MED of MGF-GKFS, R-IMM, UKF and IMM is 2.49 m, 2.84 m, 2.99 m, 3.66 m, respectively. In this distribution, the improvement of MGF-GKFS compared with R-IMM, UKF and IMM is 12.3%, 16.8% and 32.0%, respectively. From this figure, it can be observed that the 90th percentile positioning errors of MGF-GKFS are concentrated within 2.8 m; whereas, the deviation of R-IMM, UKF and IMM are concentrated within 3.4 m, 3.4 m, 4.3 m, respectively. The results indicate MGF-GKFS has a larger improvement than other methods.

**Figure 6:**
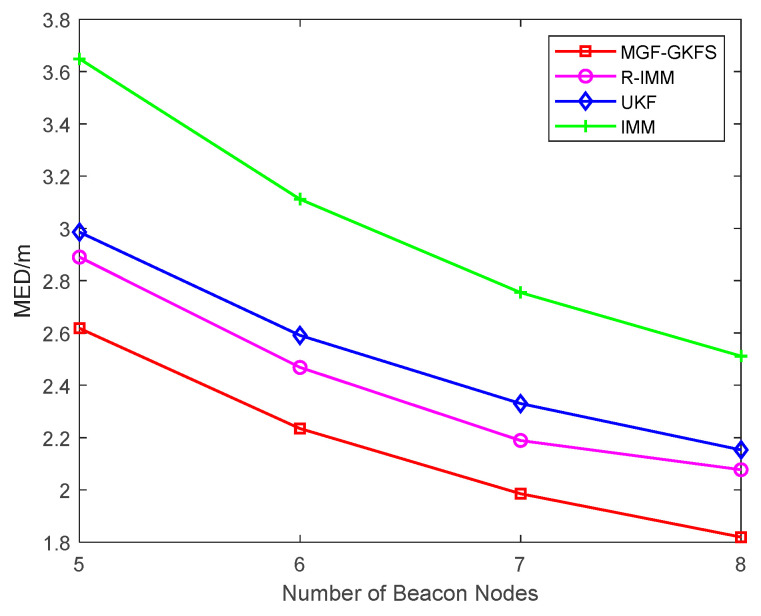
The mean error distance (MED) versus the number of beacon nodes.

To investigate the performance of MGF-GKFS with the number of beacon nodes increasing, the MED values are depicted as Figure 6 when the number changes from 5–8. It can be seen that the MED decreases when the number of beacon nodes is increasing. From this figure, MGF-GKFS obviously outperforms than other algorithms. For example, if the number is 7, the MED values of MGF-GKFS, R-IMM, UKF, IMM are, respectively, 1.98 m, 2.19 m, 2.33 m and 2.75 m.

**Figure 7:**
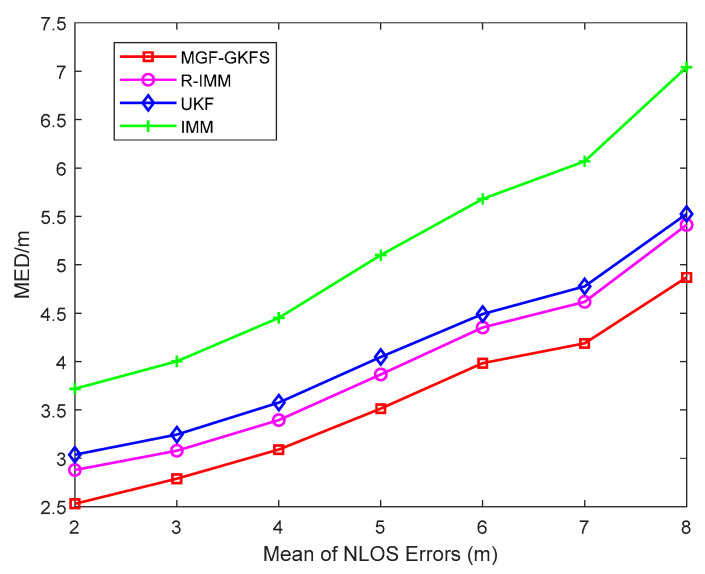
The MED versus mean of NLOS errors.

Furthermore, the trend of the MED is depicted as Figure 7 when changing the mean of NLOS errors from 2 to 8. The result shows the trend is on the rise with the mean of NLOS errors increasing. MGF-GKFS has the best performance under all the enumerated mean values of Gaussian distribution. The average MED values of MGF-GKFS, R-IMM, UKF and IMM are respectively 3.56 m, 3.94 m, 4.10 m and 5.15 m.

**Figure 8:**
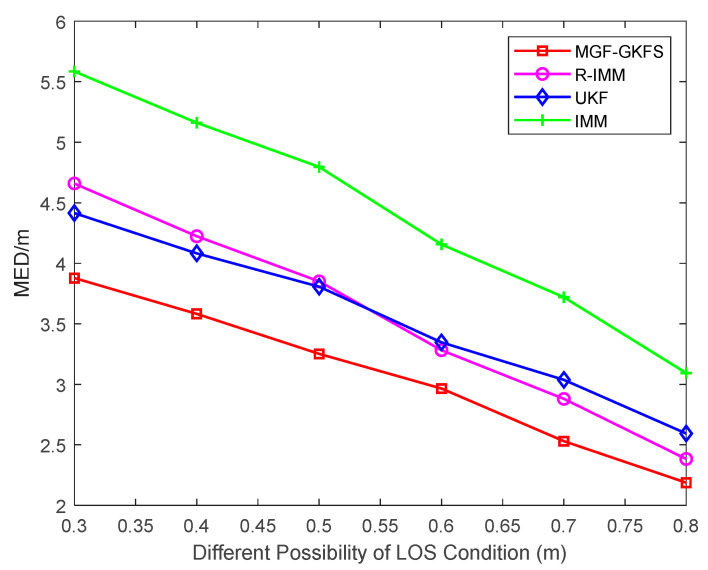
The MED versus different possibilities of LOS condition.

To further explore the positioning accuracy of MGF-GKFS, Figure 8 gives the MED results under different LOS possibilities varying from 0.3–0.8. The figure shows the MED of MGF-GKFS is generally smaller than the other three methods. The average MED values of MGF-GKFS, R-IMM, UKF and IMM are 2.86 m, 3.30 m, 3.24 m and 4.12 m, respectively. The result illustrates that MGF-GKFS is much better when coping any of the probability that NLOS errors contain.

Therefore, from the perspective of the number of beacon nodes, the various NLOS parameters and the different possibilities of the LOS environment, the conclusion can be reached that MGF-GKFS has better localization accuracy than the other three algorithms.

#### 6.1.2. Uniform Distribution

**Figure 9:**
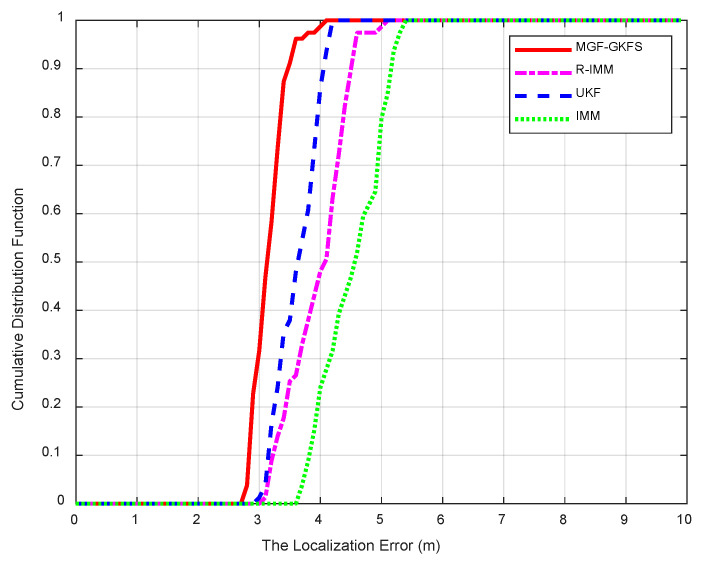
The CDF when NLOS error obeys U(3,8).

Figure 9 shows the CDF of when NLOS errors obey uniform distribution U(3,8). The average MED of MGF-GKFS, R-IMM, UKF and IMM are, respectively, 3.12 m, 3.90 m, 3.57 m, 4.46 m. The accuracy improvement compared with R-IMM, UKF and IMM are, respectively, 20.1%, 12.7% and 30.1%. It can be observed that 90-th percentile of MGF-GKFS errors is concentrated within 3.5 m whereas the deviation of R-IMM, UKF and IMM are, respectively, 4.5 m, 4.0 m and 5.1 m, meaning the improvement of our algorithm is significant.

**Figure 10:**
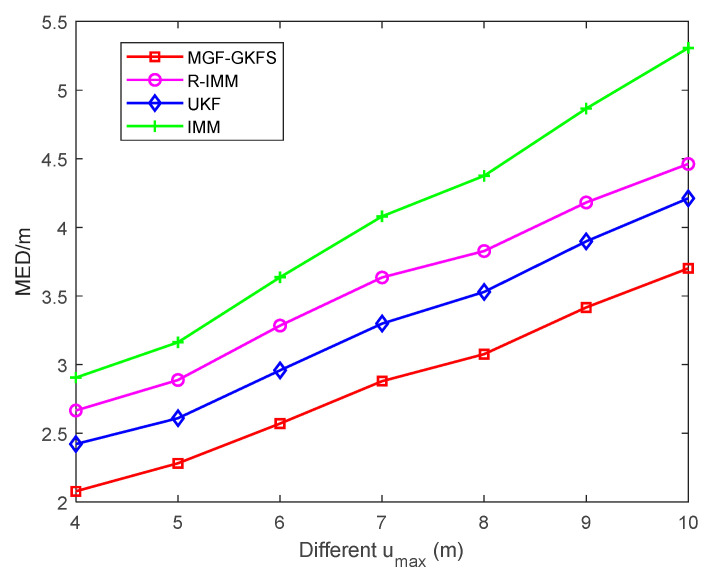
The MED versus $u_{\max}$ of uniform distribution.

Figure 10 displays the average localization errors when the parameter $u_{\max}$ changes from 4 m to 10 m. It can be clearly seen that MGF-GKFS achieves the highest localization accuracy than other methods. With the parameter $u_{\max}$ increasing, the MED values of four algorithms become larger. 

The average MED of MGF-GKFS, R-IMM, UKF and IMM are, respectively, 2.85 m, 3.56 m, 3.28 m and 4.05 m.

**Figure 11:**
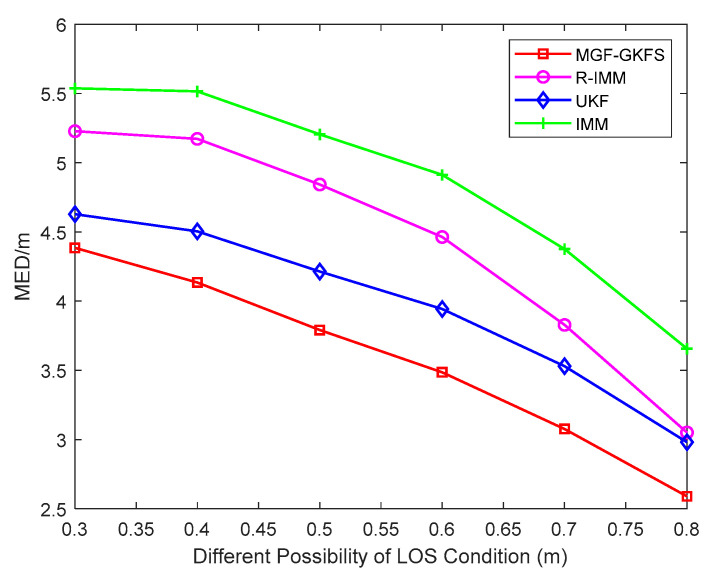
The MED versus different possibility of LOS condition.

The MED results with the LOS possibility increasing gradually are displayed as Figure 11. MGF-GKFS still maintains the highest positioning accuracy compared to the other three algorithms, which demonstrates the effectiveness of MGF-GKFS. The average MED values of MGF-GKFS, R-IMM, UKF and IMM are 3.35 m, 4.11 m, 3.73 m and 4.57 m, respectively. If the probability of LOS propagation is 0.8, the positioning accuracy of UKF and R-IMM is almost the same.

#### 6.1.3. Exponential Distribution

**Figure 12:**
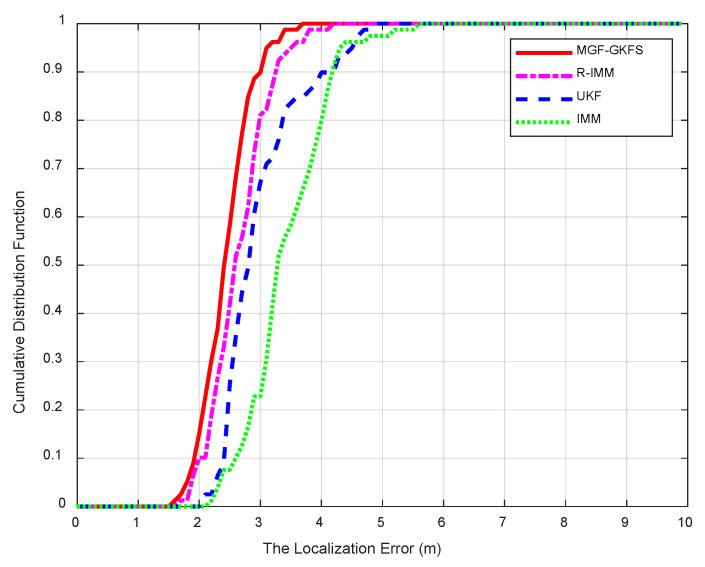
The CDF when NLOS error obeys E(3).

Figure 12 shows the CDF of localization errors obeying exponential distribution E(3). The MED values of MGF-GKFS, R-IMM, UKF and IMM are, respectively, 2.39 m, 2.61 m, 2.94 m and 3.37 m. The improvements compared to R-IMM, UKF and IMM are 8.1%, 18.5% and 29.0%, respectively, which illustrates the obvious enhancement. The 90th percentile localization errors of MGF-GKFS is concentrated within 3 m; whereas for R-IMM, UKF and IMM the 90th-percentile increases 3.3 m, 4.0 m and 4.2 m, respectively. The improvements compared with R-IMM, UKF, IMM are 9.1%, 25.0% and 28.6%, respectively. Under the exponential distribution, the performance of MGF-GKFS has been greatly improved compared to other algorithms.

**Figure 13:**
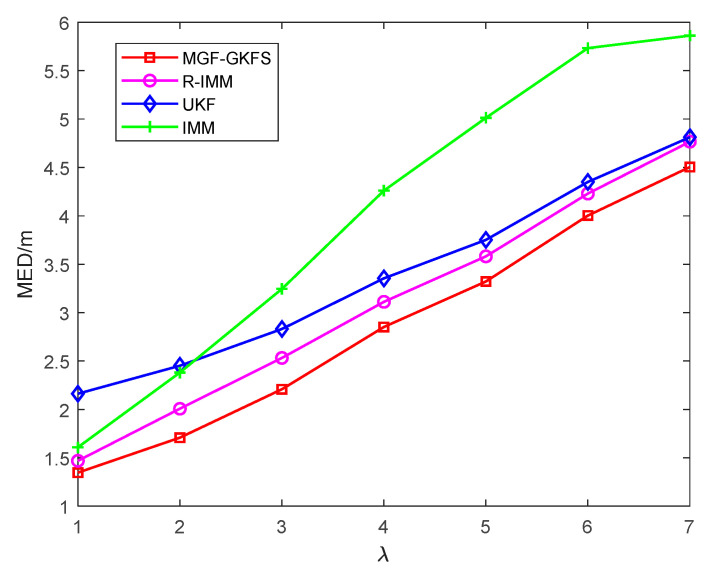
The MED versus the parameter λ.

To further investigate the robustness of MGF-GKFS, the parameter λ is varied from 1 to 7 as shown in Figure 13. When the parameter λ is 1 m, UKF has the worst performance among the four algorithms, and MGF-GKFS, R-IMM, IMM almost have the same localization accuracy. IMM is very sensitive to the NLOS errors changing, in which the positioning error rises very quickly as the parameter increases. By contrast, the performance of MGF-GKFS under different NLOS errors is stably better than R-IMM, UKF and IMM. It achieves the average MED of 2.93 m while3.09 m for R-IMM, 3.38 m for UKF and 4.01 m for IMM.

### 6.2. Experiment Results

An experiment is conducted to validate the performance of our algorithm in an indoor environment. Ultra-wideband (UWB) module is adopted in this paper to send measurement signal to the mobile node and transmit the received non-sinusoidal narrow pulse (less than 1 ns) to distance information. The UWB signal is resistant to channel fading and the multipath effect can be effectively decomposed by UWB receivers, so UWB can achieve high localization accuracy at the centimeter level in indoor localization.

The model of UWB module in this experiment is D-DWM-PG1.7V, which contains four modules: the DWM 100 module, the STM32F1 microcontroller unit (MCU), the USB serial port and the SW download port. We use transistor transistor logic (TTL) serial port for download and communication, USB interface for power supply, TTL serial port to connect to PC. We use two-dimensional positioning method to construct the scenario. The serial port of the main base station is connected to the computer. The baud rate is set to 115,200 bps. The secondary base stations are randomly placed in different locations of the room. The mobile node is tagged so that the base station can identify it. The MODBUS-ID is set to 1.

**Figure 14:**
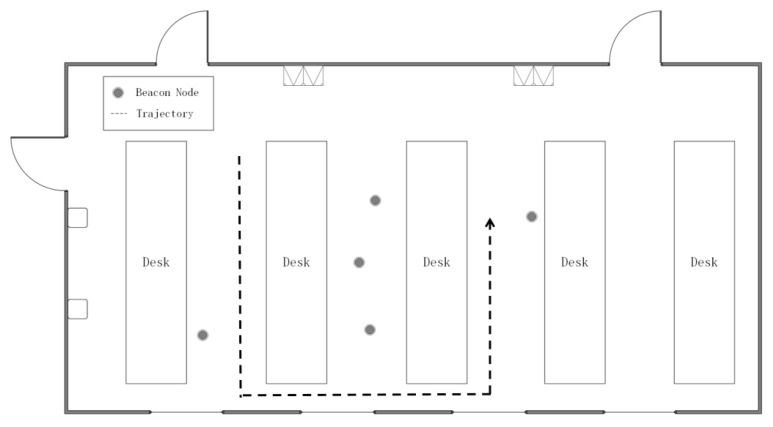
The floor plan of the experiment scenario in an indoor environment.

Five beacon nodes are randomly deployed in a 15 × 8 m^2^ room. The height of all tables is 1.2 m. The beacon nodes and the mobile node is set up to 1.1 m above the ground. The movement trajectory of the mobile node is shown as Figure 14. The frequency of measurement is 20 Hz. Until now, we have successfully set up a LOS/NLOS mixed environment according to the above steps.

**Figure 15:**
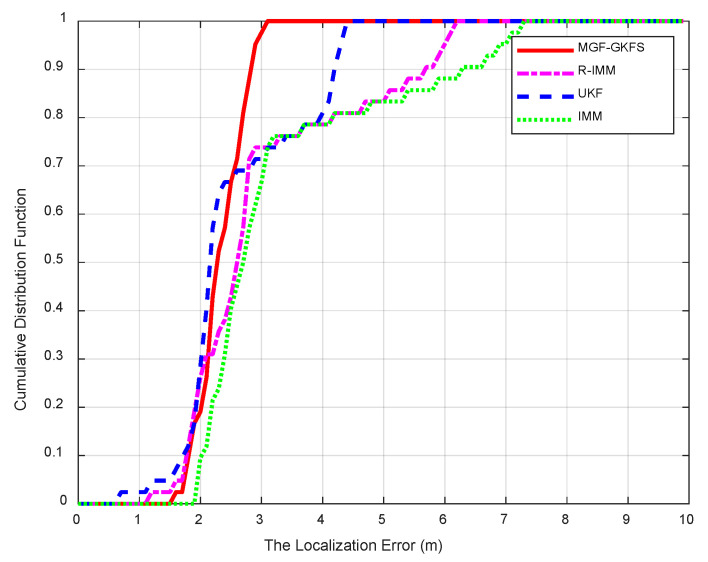
The CDF versus the localization errors.

The CDF of positioning errors is shown as Figure 15. The average MED values of MGF-GKFS, R-IMM, UKF and IMM are, respectively, 2.2699 m, 2.9285 m, 2.5087 m and 3.2078 m. The improvements of MGF-GKFS compared to R-IMM, UKF and IMM are 22.4%, 9.5% and 29.2%, which illustrates obvious enhancement compared with the three other algorithms.

Table 3 lists the processing time of each algorithm in actual scenario conducted in this section. The platform configuration was as follows. The computer operating system was Windows 10 Professional. The version of processor was Intel(R) Core(TM) i5-7200U @ 2.50GHz and 8.00 GB RAM. The simulation platform was MATLAB R2019a.

### 6.3. Computational Analysis

The time complexity is highly relative to the number of multiplications. According to the pseudocode given in the first few sections, the number of multiplications could be counted. The convergence time of the EM algorithm plays an important role in the total complexity. The total multiplication numbers of GKF and UKF is 80, without considering GMM. One iteration of GMM needs 18 multiplications. Assume that after $n_{k1}$ times of iterations, EM algorithm convergences. Therefore the total number of multiplications is $80+18n_{k1}$. In R-IMM, the computational complexity depends on the rate of convergence of the robust regression algorithms [29]. The bounded score function is used to update the state vector. When the distance between the last state and the current state (i.e., second norm) is less than the threshold ε, the iteration process stops. Assume that the iteration times of a robust regression algorithm is $n_{k2}$. The number of multiplications is compared in Table 3.

From this table, it can be observed that the computational complexity is strongly related to the iteration times of EM algorithms or robust regression algorithms. However the iteration times depend on the current environment (i.e., the degree of interference under NLOS errors). In Table 4, the total computation time is listed when algorithms ran in the real scene of Section 6.2. It can also reflect the computational complexity. Because of the introduction of the EM algorithm, it takes some time for the likelihood function to converge. Therefore, MGF-GKFS is inferior to other algorithms in terms of time performance. In future work, we will investigate some effective methods to reduce the large amount of calculation caused by EM, for example, expanding the time interval for refitting the mixed Gaussian distribution or properly discarding points that are too far away from the present moment.

The space complexity of MGF-GKFS is the same as the other algorithms. All the algorithms presented in this paper are based on distance filtering. That is to say, the beacon nodes only need to store the current measurement and forward it to the task-management center.

## 7. Conclusions

In this paper, we propose a novel indoor localization method in a mixed LOS/NLOS environment based on grey Kalman filter, mixture Gaussian fitting and unscented Kalman filter. The proposed GKF can effectively mitigate the process noise and alleviate the NLOS error, and its accuracy is significantly higher than IMM. GKF makes the difference between the measured value and the true value closer to the NLOS error, ensuring the accuracy of the residual calculation, which lays a solid foundation for correct clustering in MGF. A soft NLOS identification method is presented to detect the propagation environment and can give the degree of membership accurately. For the LOS case, the filtered result of GKF can be used as the final value. For the NLOS case, UKF with a large covariance matrix is used to further mitigate the NLOS error. Multiple filters are combined with membership. Full-scale simulations and experiments are discussed to ensure the conclusion is persuasive, illustrating MGF-GKFS is obviously better than R-IMM, UKF and IMM algorithms in a complicated indoor environment.

## Figures and Tables

**Table 1:** Notations.

Symbol	Explanation	Symbol	Explanation
*M*	the number of beacon nodes	*N*	total time
$d_{i}^{\left(0\right)}(k)$	the measured distance	$\sigma_{i}^{2}$	the variance of sensor noise
${\dot{d}}_{i}^{\left(0\right)}(k)$	the measured velocity	F	the initial state transition matrix
${\hat{F}}_{i}$	the updated state transition matrix in grey Kalman filter(GKF)	$x_{i}(k)$	the state vector
$z_{i}(k)$	the measurement vector	H	observation matrix
$\nu_{i}(k)$	process noise	$w_{i}(k)$	measurement noise
$l_{c}$	constant series length of predicting points	${\tilde{d}}_{i}^{\left(0\right)}(k)$	the filtered distance of GKF
$y_{i,k}^{\left(0\right)}(n)$	the *n*-th element of original series of distance	$y_{i,k}^{\left(0\right)}$	original series of distance
$y_{i,k}^{\left(1\right)}(n)$	the *n*-th element of accumulative series of distance	$y_{i,k}^{\left(1\right)}$	accumulative series of distance
$a_{i,k}$	development coefficient	$u_{i,k}$	grey effect coefficient
${\hat{y}}_{i,k}^{\left(0\right)}(n)$	the *n*-th element of predicted series of distance	${\hat{y}}_{i,k}^{\left(0\right)}$	predicted series of distance
${\hat{y}}_{i,k}^{\left(1\right)}(n)$	the *n*-th element of predicted accumulative series of distance	${\hat{y}}_{i,k}^{\left(1\right)}$	predicted accumulative series
$v_{i,k}^{\left(1\right)}$	accumulative series value of speed	$P_{i}(k)$	the variance matrix of the state vector
$\zeta_{i}(k)$	the residual value between the measured distance and the filtered distance processed by GKF	$\mu_{j}$	the mean value of *j*-th single Gaussian distribution
$L_{i}^{k}(\theta )$	the likelihood function of EM algorithm	*G*	the number of single Gaussian distribution
$\theta_{j}$	the distribution parameter set of *j*-th single Gaussian distribution	$Y_{j}^{t}(\zeta_{i}(k))$	the probability function that the residual value belongs to *j*-th Gaussian distribution at iteration *t*
$dis_{LOS}^{i}(k)$	the Euclidean distance between the current residual value and the small mean Gaussian distribution centre	$dis_{NLOS}^{i}(k)$	the Euclidean distance between the current residual value and the big mean Gaussian distribution centre
$p_{LOS}^{i}(k)$	the probability of LOS condition	$p_{NLOS}^{i}(k)$	the probability of NLOS condition
${\tilde{d}}_{i}(k)$	the final filtered distance	${\tilde{d}}_{i}^{\left(UKF\right)}(k)$	the filtered distance of UKF

**Table 2:** The default parameters.

Description	Notation	Default Values
The number of beacon nodes	*M*	5
The probability of line of sight (LOS )condition	φ	0.7
The sensor noise	$\sigma_{i}$	1
The total time of target moving	*K*	80
The number of Monte Carlo running	$T_{n}$	1000
Gaussian distribution	$N(\mu_{NLOS},\sigma_{NLOS}^{2})$	$N(2,4^{2})$
Uniform distribution	$U(u_{\min},u_{\max})$	U(3,8)
Exponential distribution	E(λ)	E(3)

**Table 3:** Total number of multiplications.

Algorithm	Multiplications
MGF-GKFS	$80+18n_{k1}$
R-IMM	$56+49n_{k2}$
UKF	59
IMM	98

**Table 4:** Total computational time.

Algorithm	Running Time/s
MGF-GKFS	0.0521
R-IMM	0.0221
UKF	0.0032
IMM	0.0128
